# Are Noradrenergic Transmission Reducing Drugs Antidepressants?

**DOI:** 10.3389/fnbeh.2021.673634

**Published:** 2021-09-30

**Authors:** Paul J. Fitzgerald

**Affiliations:** Department of Psychiatry, University of Michigan, Ann Arbor, MI, United States

**Keywords:** norepinephrine, noradrenaline, serotonin, SSRI (selective serotonergic reuptake inhibitors), propranolol, clonidine, guanfacine, prazosin

## Abstract

Major depressive disorder (MDD) remains a significant public health problem worldwide, and revised treatment strategies are therefore urgently needed, including the creation of novel antidepressant compounds or using existing molecular entities in new ways. Etiologic theories of MDD from decades ago have suggested that synaptic deficiencies of monoaminergic neurotransmitters play a causative role in this neuropsychiatric disorder, and that boosting monoamines with drugs such as SSRIs, SNRIs, TCAs, and MAOIs has antidepressant effects and in some individuals can even induce hypomania or mania. While other factors, such as various intracellular molecular pathways and hippocampal neurogenesis, undoubtedly also play a role in MDD, monoaminergic boosting drugs nonetheless have clearly demonstrated antidepressant properties. There is also, however, a body of studies in the preclinical literature suggesting that monoaminergic transmission *reducing* drugs, including noradrenergic ones, also have antidepressant-like behavioral properties in rodents. Given that there is increasing evidence that the monoamines have u-shaped or Janus-faced dose-response properties, in which a mid-range value is “optimal” in a variety of behavioral and physiological processes, it is plausible that either too much or too little synaptic norepinephrine in key circuits may exacerbate MDD in some individuals. Here we briefly review rodent depression-related behavioral data, focusing on the forced swim test, from three major classes of noradrenergic transmission reducing drugs (alpha2 agonists, beta blockers, alpha1 antagonists), and find much support for the hypothesis that they have antidepressant-like properties. Whether these drugs are antidepressants in human subjects remains to be determined.

## Introduction

Despite intensive efforts by commercial and academic researchers for many decades, major depressive disorder (MDD) remains a significant source of morbidity and mortality throughout the world (Chen et al., 2017; Schmaal et al., 2017; Hasin et al., 2018; Ingram et al., 2020). Many individuals who experience MDD do not respond completely, or in some cases at all, to existing pharmacological or behavioral treatment modalities, leaving a need for new approaches (Mitchell, 2004; Ruhé et al., 2006; Ulrich et al., 2020). In addition to the demand for improved psychotherapeutic treatments, the field would benefit from the creation of novel pharmacological agents or the repurposing of existing compounds that may, perhaps unexpectedly, have beneficial properties in the treatment of MDD (Ebada, 2017; Demin et al., 2019).

Following the discovery of some of today’s widely used antidepressants (MAOIs, TCAs) in the mid-20th century, monoaminergic theories on the etiology of MDD were put forth, suggesting that diminished brain levels of serotonin, norepinephrine (NE), and dopamine are a causative factor in the disorder (Schildkbaut, 1965; Coppen, 1967; Janowsky et al., 1972). In the decades since then, it has become increasingly clear that a number of intracellular molecular pathways (which undoubtedly interact with the extracellular monoamines) also play a role in MDD and the physiological and behavioral responses to antidepressant drugs (Vaidya and Duman, 2001; Tanis and Duman, 2007; Miller et al., 2009; Wohleb et al., 2016), although the continued medical use of antidepressants that boost synaptic monoamines (including SSRIs, SNRIs, NDRIs, TCAs, MAOIs) reinforces the clinical utility of this approach.

For these reasons, it may be surprising to note that in the preclinical literature there is also a significant body of studies suggesting that noradrenergic transmission *reducing* drugs, such as the alpha2 agonist clonidine, exhibit antidepressant-like behavioral properties under a variety of experimental conditions. This may be a surprising finding since a number of the monoaminergic theories of MDD suggest that elevated monoamines should produce mania or hypomania (Schildkbaut, 1965; Coppen, 1967; Janowsky et al., 1972), and by inference transmission reducing drugs may have mood-stabilizing properties but not necessarily be antidepressants. However, a growing body of evidence suggests that endogenous serotonin, NE, and dopamine have u-shaped or Janus-faced dose-response properties for a range of behaviors, wherein too much or too little signaling may be pathological (Baldi and Bucherelli, 2005; Arnsten, 2007; Vijayraghavan et al., 2007; Giustino et al., 2016; Giustino and Maren, 2018; Groft et al., 2019). In this scenario, perhaps a non-optimal (i.e., decreased or elevated) synaptic concentration of each monoamine may result in MDD, at least in some individuals with the disorder.

Below we briefly review rodent preclinical findings on the depression-related behavioral effects of three major classes of noradrenergic transmission reducing drugs: alpha2 agonists, beta blockers, and alpha1 antagonists. We focus on three major behavioral assays: the forced swim test (FST), the tail suspension test (TST), and the sucrose preference test. We conducted a PubMed database search using the following terms (February 7, 2021): clonidine/guanfacine/dexmedetomidine/propranolol/carvedilol/nebivolol/metoprolol/atenolol/prazosin/“beta blocker(s)”/alpha1/alpha2/beta1/beta2/beta3 +“forced swim”/“forced swimming”/“tail suspension”/“sucrose preference”/antidepressant-like/depression-like. This literature search yielded a total of 489 publications. Forty-eight were judged to be relevant articles that included data with at least one of the above types of drugs (alpha2 agonists, beta blockers, alpha1 antagonists), in mice or rats that were exposed to at least one of the above three behavioral assays (FST, TST, sucrose preference). To be included, these papers had to be published in the English language, and the 48 that met these criteria are further described in Table 1. There was no limit set on how long ago the papers were published. We did not focus on studies that investigated the interaction between natural products or compounds and these noradrenergic agents.

**Table 1:** Summary of antidepressant-related effects of noradrenergic transmission reducing drugs.

Publication	Species	Strain	Sex	Primary Drug	Secondary Drug	Dose (mg/kg)	Route	Repeats	Time Delay	Stress	Test	Effect
O’Neill et al. (2001)	M	BKTO	F	clonidine	none	0.25	s.c.	0	30 min	none	FS	dec imm
				clonidine	none	0.5	s.c.	0	30 min	none	FS	dec imm
				clonidine	none	1	s.c.	0	30 min	none	FS	dec imm
Malikowska et al. (2017)	M	CD-1	M	clonidine	none	0.1	i.p.	0	60 min	none	FS	dec imm
	M	CD-1	M	clonidine	none	0.1	i.p.	0	60 min	24 h after SPS	FS	n.s.
Asakura et al. (1993)	M	ddY	M	clonidine	none	0.03	i.p.	0	30 min	none	FS	n.s.
				clonidine	none	0.1	i.p.	0	30 min	none	FS	n.s.
				clonidine	none	0.3	i.p.	0	30 min	none	FS	inc swim
				clonidine	none	0.03	i.p.	0	30 min	48 h soc isol	FS	n.s.
				clonidine	none	0.1	i.p.	0	30 min	48 h soc isol	FS	n.s.
				clonidine	none	0.3	i.p.	0	30 min	48 h soc isol	FS	inc swim
Asakura et al. (1994)	M	ddY	M	clonidine	none	0.03	i.p.	0	30 min	none	FS	n.s.
				clonidine	none	0.1	i.p.	0	30 min	none	FS	n.s.
				clonidine	none	0.3	i.p.	0	30 min	none	FS	inc swim
				clonidine	none	1	i.p.	0	30 min	none	FS	inc swim
				clonidine	none	0.03	i.p.	0	30 min	48 h soc isol	FS	n.s.
				clonidine	none	0.1	i.p.	0	30 min	48 h soc isol	FS	n.s.
				clonidine	none	0.3	i.p.	0	30 min	48 h soc isol	FS	inc swim
				clonidine	none	1	i.p.	0	30 min	48 h soc isol	FS	inc swim
Masuda et al. (2001)	M	ddY	M	clonidine	none	0.004	i.p.	0	45 min	none	FS	inc clim
				clonidine	none	0.02	i.p.	0	45 min	none	FS	inc clim
				clonidine	none	0.1	i.p.	0	45 min	none	FS	inc clim
Kaster et al. (2007)	M	Swiss	F	clonidine	none	0.06	i.p.	0	60 min	none	FS	n.s.
Kotagale et al. (2013)	M	Swiss	M	clonidine	none	0.015	i.p.	0	30 min	none	FS	n.s.
				clonidine	bupropion	0.015, 5	i.p.	0	30 min	none	FS	dec imm
Hascoät et al. (1991)	M	Swiss	M	clonidine	none	0.06	i.p.	0	30 min	none	FS	n.s.
				clonidine	none	0.125	i.p.	0	30 min	none	FS	inc mob
				clonidine	none	0.25	i.p.	0	30 min	none	FS	n.s.
				clonidine	none	0.5	i.p.	0	30 min	none	FS	inc mob
				clonidine	none	1	i.p.	0	30 min	none	FS	inc mob
				clonidine	none	0.06	i.p.	0	30 min	none	TS	n.s.
				clonidine	none	0.125	i.p.	0	30 min	none	TS	n.s.
				clonidine	none	0.25	i.p.	0	30 min	none	TS	dec imm
				clonidine	none	0.5	i.p.	0	30 min	none	TS	n.s.
				clonidine	none	1	i.p.	0	30 min	none	TS	n.s.
Malinge et al. (1988)	M	Swiss	M	clonidine	none	0.015	i.p.	0	30 min	none	FS	n.s.
				clonidine	none	0.06	i.p.	0	30 min	none	FS	inc mob
				clonidine	none	0.25	i.p.	0	30 min	none	FS	inc mob
				clonidine	none	1	i.p.	0	30 min	none	FS	inc mob
				clonidine	none	4	i.p.	0	30 min	none	FS	inc mob
				clonidine	none	16	i.p.	0	30 min	none	FS	inc mob
				clonidine	imipramine	0.06, 4	i.p.	0	30 min	none	FS	dec imm
				clonidine	amitriptyline	0.06, 1	i.p.	0	30 min	none	FS	dec imm
				clonidine	maprotiline	0.06, 8	i.p.	0	30 min	none	FS	dec imm
				clonidine	mianserin	0.06, 4	i.p.	0	30 min	none	FS	dec imm
				clonidine	viloxazine	0.06, 2	i.p.	0	30 min	none	FS	dec imm
				clonidine	citalopram	0.06, 2	i.p.	0	30 min	none	FS	dec imm
				clonidine	indalpine	0.06, 4	i.p.	0	30 min	none	FS	dec imm
				clonidine	fluvoxamine	0.06, 4	i.p.	0	30 min	none	FS	dec imm
				clonidine	inprindole	0.06, 32	i.p.	0	30 min	none	FS	dec imm
				clonidine	nialamide	0.06, 32	i.p.	0	30 min	none	FS	dec imm
Bourin et al. (1991)	M	Swiss	M	clonidine	imipramine	0.1, 8	i.p.	0	30 min	none	FS	dec imm
				clonidine	amitriptyline	0.1, 2	i.p.	0	30 min	none	FS	dec imm
				clonidine	maprotiline	0.1, 8	i.p.	0	30 min	none	FS	dec imm
				clonidine	citalopram	0.1, 4	i.p.	0	30 min	none	FS	dec imm
				clonidine	fluvoxamine	0.1, 8	i.p.	0	30 min	none	FS	dec imm
				clonidine	paroxetine	0.1, 8	i.p.	0	30 min	none	FS	dec imm
Bourin et al. (1996)	M	Swiss	M	clonidine	imipramine	0.06, 4	i.p.	0	30 min	none	FS	dec imm
				clonidine	fluoxetine	0.06, 2	i.p.	0	30 min	none	FS	dec imm
				clonidine	trazodone	0.06, 0.5	i.p.	0	30 min	none	FS	dec imm
				clonidine	mianserin	0.06, 4	i.p.	0	30 min	none	FS	dec imm
				clonidine	gepirone	0.06, 4	i.p.	0	30 min	none	FS	dec imm
Bourin et al. (2002)	M	Swiss	M	clonidine	tranylcypromine	0.06, 0.5	i.p.	0	30 min	none	FS	dec imm
				clonidine	phenelzine	0.06, 8	i.p.	0	30 min	none	FS	dec imm
Hascoet et al. (1994)	M	Swiss	M	clonidine	8-OH-DPAT	0.1, 0.5	i.p.	0	30 min	none	FS	n.s.
				clonidine	gepirone	0.1, 4	i.p.	0	30 min	none	FS	dec imm
				clonidine	ipsapirone	0.1, 1	i.p.	0	30 min	none	FS	dec imm
Redrobe and Bourin (1997)	M	Swiss	M	clonidine	imipramine	0.06, 4	i.p.	0	30 min	none	TS	dec imm
				clonidine	fluoxetine	0.06, 2	i.p.	0	30 min	none	TS	dec imm
				clonidine	trazodone	0.06, 0.5	i.p.	0	30 min	none	TS	dec imm
				clonidine	mianserin	0.06, 4	i.p.	0	30 min	none	TS	dec imm
				clonidine	iprindole	0.06, 32	i.p.	0	30 min	none	TS	n.s.
				clonidine	ritanserin	0.06, 0.5	i.p.	0	30 min	none	TS	dec imm
				clonidine	ipsapirone	0.06, 1	i.p.	0	30 min	none	TS	dec imm
Redrobe and Bourin (1998)	M	Swiss	M	clonidine	8-OH-DPAT	0.06, 1	i.p.	0	30 min	none	FS	dec imm
				clonidine	ritanserin	0.06, 4	i.p.	0	30 min	none	FS	dec imm
				clonidine	ketanserin	0.06, 8	i.p.	0	30 min	none	FS	n.s.
Taksande et al. (2009)	M	Swiss	M	clonidine	none	0.015	i.p.	0	30 min	none	FS	n.s.
				clonidine	none	0.03	i.p.	0	30 min	none	FS	n.s.
				clonidine	none	0.06	i.p.	0	30 min	none	FS	dec imm
				clonidine	imipramine	0.015, 2.5	i.p.	0	30 min	none	FS	n.s.
				clonidine	fluoxetine	0.015, 2.5	i.p.	0	30 min	none	FS	dec imm
				clonidine	paroxetine	0.015, 2.5	i.p.	0	30 min	none	FS	dec imm
Zeidan et al. (2007)	M	Swiss	M, F	clonidine	none	0.06	i.p.	0	30 min	none	FS	n.s.
				clonidine	agmatine	0.06, 0.001	i.p.	0	30 min	none	FS	dec imm
Ferrari et al. (1991)	M	Swiss	M	clonidine	none	0.075	i.p.	0	25 min	none	TS	inc imm
				clonidine	none	0.15	i.p.	0	25 min	none	TS	inc imm
Parale and Kulkarni (1986)	M	Wist	M	clonidine	none	0.05	i.p.	0	15 min	none	FS	inc imm
				clonidine	none	0.15	i.p.	0	15 min	none	FS	inc imm
				clonidine	none	0.5	i.p.	0	15 min	none	FS	inc imm
Evangelista et al. (1987)	R	CD-COBS	M	clonidine	none	0.1	i.p.	0	30 min	none	FS	n.s.
Cervo and Samanin (1991)	R	S-D	M	clonidine	none	0.05	i.p.	0	30 min	none	FS	n.s.
				clonidine	none	0.1	i.p.	0	30 min	none	FS	n.s.
				clonidine	none	0.5	i.p.	0	30 min	none	FS	n.s.
				clonidine	none	0.05	i.p.	2	30 min	none	FS	dec imm
				clonidine	none	0.1	i.p.	2	30 min	none	FS	dec imm
				clonidine	none	0.5	i.p.	2	30 min	none	FS	dec imm
				clonidine	none	0.1	i.p.	b.i.d. for 15 days	30 min	none	FS	n.s.
Cervo et al. (1992)	R	S-D	M	clonidine	none	0.1	i.p.	2	30 min	none	FS	dec imm
Kitada et al. (1983)	R	S-D	M	clonidine	none	0.3	s.c.	2	30 min	none	FS	n.s.
				clonidine	desipramine	0.3, 20	s.c., i.p.	2	30 min	none	FS	inc imm
Rénéric et al. (2002)	R	S-D	M	clonidine	none	0.005	i.p.	2	60 min	none	FS	inc swim
				clonidine	none	0.01	i.p.	2	60 min	none	FS	n.s.
				clonidine	none	0.02	i.p.	2	60 min	none	FS	n.s.
				clonidine	none	0.2	i.p.	2	60 min	none	FS	inc clim
Skrebuhhova et al. (1999)	R	Wist	M	clonidine	none	0.1	i.p.	1	30 min	none	FS	n.s.
				clonidine	none	1	i.p.	1	30 min	none	FS	dec imm
				clonidine	desipramine	0.1, 10	i.p.	1	15 min	none	FS	dec imm
Antkiewicz-Michaluk et al. (2017)	R	Wist	M	clonidine	none	0.1	i.p	0	60 min	none	FS	inc clim
Mineur et al. (2015)	M	C57	M, F	guanfacine	none	0.05	i.p.	0	30 min	none	FS	n.s.
				guanfacine	none	0.1	i.p.	0	30 min	none	FS	n.s.
				guanfacine	none	0.15	i.p.	0	30 min	none	FS	dec imm
				guanfacine	none	0.3	i.p.	0	30 min	none	FS	n.s.
				guanfacine	none	0.05	i.p.	q.d. for 15 days	approx 24 h	none	FS	n.s.
				guanfacine	none	0.1	i.p.	q.d. for 15 days	approx 24 h	none	FS	dec imm
				guanfacine	none	0.15	i.p.	q.d. for 15 days	approx 24 h	none	FS	dec imm
				guanfacine	none	0.3	i.p.	q.d. for 15 days	approx 24 h	none	FS	n.s.
Mineur et al. (2018)	M	C57	M, F	guanfacine	none	0.15	i.p.	0	30 min	none	FS	dec imm
				guanfacine	none	0.15	i.p.	0	30 min	none	TS	dec imm
Parale and Kulkarni (1986)	M	Wist	M	guanfacine	none	0.15	i.p.	0	15 min	none	FS	inc imm
Stone et al. (2011)	M	S-W	M	dexmedetomidine	none	0.04 nmol	i.c.v.	0	5 min	none	TS	dec imm
				dexmedetomidine	none	0.1 nmol	i.c.v.	0	5 min	none	TS	dec imm
Al-Tubuly et al. (2008)	M	albino	NS	propranolol	none	1	i.p.	0	60 min	none	FS	dec latency to imm
				propranolol	imipramine	1, 10	i.p.	0	60 min	none	FS	dec latency to imm
Sekio and Seki (2015)	M	CD-1	M	propranolol	LPS	5 μl 400 mM	i.c.v.	0	24 h	none	FS	n.s.
				propranolol	LPS	5 μl 400 mM	i.c.v.	0	24 h	none	TS	n.s.
Zhang et al. (2009)	M	FVB	M	propranolol	none	2.5	i.p.	0	45 min	none	FS	n.s.
				propranolol	desipramine	2.5, 20	i.p.	0	30 min	none	FS	n.s.
Gu et al. (2012)	M	ICR	M	propranolol	none	5	i.p.	0	120 min	none	TS	n.s.
Teste et al. (1990)	M	NMRI	M	propranolol	none	0.12	i.p.	0	60 min	none	TS	n.s.
				propranolol	none	0.5	i.p.	0	60 min	none	TS	n.s.
				propranolol	none	2	i.p.	0	60 min	none	TS	n.s.
				propranolol	none	8	i.p.	0	60 min	none	TS	n.s.
Pesarico et al. (2014)	M	Swiss	M	propranolol	none	2	i.p.	0	45 min	none	FS	n.s.
Evangelista et al. (1987)	R	CD-COBS	M	propranolol	none	5	i.p.	0	120 min	none	FS	n.s.
Abel and Hannigan (1994)	R	F344	M	propranolol	none	1	i.p.	0	60 min	none	FS	n.s.
				propranolol	none	3	i.p.	0	60 min	none	FS	inc imm
				propranolol	none	5	i.p.	0	60 min	none	FS	inc imm
				propranolol	none	1	i.p.	q.d. for 10 days	60 min	none	FS	n.s.
				propranolol	none	3	i.p.	q.d. for 10 days	60 min	none	FS	n.s.
				propranolol	none	5	i.p.	q.d. for 10 days	60 min	none	FS	n.s.
Finnegan et al. (1987)	R	S-D	M	propranolol	none	5	i.p.	q.d. for 7 days	24 h	none	FS	n.s.
Zaidi et al. (2020)	R	S-D	M	propranolol	none	50/day	in water	given for 36 days	9 days	none	FS	n.s.
				propranolol	none	50/day	in water	given for 36 days	9 days	soc defeat	FS	dec imm
Aisa et al. (2008)	R	Wist	F	propranolol	none	2	s.c.	0	60 min	mat sep	FS	dec imm
Zaidi et al. (2020)	R	S-D	M	nadolol	none	18/day	in chow	given for 36 days	9 days	none	FS	n.s.
				nadolol	none	18/day	in chow	given for 36 days	9 days	soc defeat	FS	dec imm
Park et al. (2012)	M	C57	M	butoxamine	none	5	i.p.	1	30 min	none	FS	n.s.
Al-Tubuly et al. (2008)	M	albino	NS	atenolol	none	5	i.p.	0	60 min	none	FS	inc latency to imm
				atenolol	imipramine	5, 10	i.p.	0	60 min	none	FS	n.s.
Stone and Quartermain (1999)	M	S-W	M	betaxolol	none	5	i.p.	0	20 min	none	TS	n.s.
				betaxolol	none	20	i.p.	0	20 min	none	TS	n.s.
Detke et al. (1995)	R	S-D	M	betaxolol	none	10	s.c.	2	60 min	none	FS	n.s.
				betaxolol	8-OH-DPAT	10, 0.5	s.c.	2	60 min	none	FS	n.s.
Zaidi et al. (2020)	R	S-D	M	bisoprolol	none	15/day	in water	given for 36 days	9 days	none	FS	n.s.
				bisoprolol	none	15/day	in water	given for 36 days	9 days	soc defeat	FS	n.s.
Park et al. (2012)	M	C57	M	metoprolol	none	10	i.p.	1	30 min	none	FS	n.s.
Al-Tubuly et al. (2008)	M	albino	NS	prazosin	none	5	i.p.	0	60 min	none	FS	dec latency to imm
			NS	prazosin	imipramine	5, 10	i.p.	0	60 min	none	FS	inc latency to imm
Sekio and Seki (2015)	M	CD-1	M	prazosin	LPS	5 μl 70 mM	i.c.v.	0	24 h	none	FS	dec imm
				prazosin	LPS	5 μl 70 mM	i.c.v.	0	24 h	none	TS	dec imm
Kurosawa et al. (2016)	M	CD-1	M	prazosin	inflammatory cytokines	280 μg	i.c.v.	0	24 h	none	FS	dec imm
				prazosin	inflammatory cytokines	280 μg	i.c.v.	0	24 h	none	TS	dec imm
Sugimoto et al. (2011)	M	DBA/2Cr	M	prazosin	none	1	i.p.	0	60 min	none	FS	n.s.
	M	DBA/2Cr	M	prazosin	none	5	i.p.	0	60 min	none	FS	n.s.
	M	DBA/2Cr	M	prazosin	paroxetine	1, 5	i.p.	0	30 min	none	FS	n.s.
	M	DBA/2Cr	M	prazosin	paroxetine	5, 5	i.p.	0	30 min	none	FS	inc imm
	M	ICR	M	prazosin	none	1	i.p.	0	60 min	none	FS	n.s.
	M	ICR	M	prazosin	none	5	i.p.	0	60 min	none	FS	n.s.
	M	ICR	M	prazosin	paroxetine	1, 5	i.p.	0	30 min	none	FS	n.s.
	M	ICR	M	prazosin	paroxetine	5, 5	i.p.	0	30 min	none	FS	inc imm
Gu et al. (2012)	M	ICR	M	prazosin	none	0.0625	i.p.	0	120 min	none	TS	n.s.
Teste et al. (1990)	M	NMRI	M	prazosin	none	1	i.p.	0	60 min	none	TS	n.s.
				prazosin	none	2	i.p.	0	60 min	none	TS	n.s.
				prazosin	none	4	i.p.	0	60 min	none	TS	n.s.
				prazosin	none	8	i.p.	0	60 min	none	TS	n.s.
Pesarico et al. (2014)	M	Swiss	M	prazosin	none	1	i.p.	0	45 min	none	FS	n.s.
Ribeiro and Pupo (2015)	M	Swiss	M	prazosin	none	0.5	i.p.	0	30 min	none	TS	n.s.
				prazosin	none	1	i.p.	0	30 min	none	TS	inc imm
				prazosin	imipramine	0.5, 32	i.p.	0	30 min	none	TS	inc imm
				prazosin	imipramine	1, 32	i.p.	0	30 min	none	TS	inc imm
Kaster et al. (2007)	M	Swiss	F	prazosin	none	1	i.p.	0	60 min	none	FS	n.s.
Hascoät et al., 1991	M	Swiss	M	prazosin	none	0.25	i.p.	0	30 min	none	FS	n.s.
				prazosin	none	0.5	i.p.	0	30 min	none	FS	n.s.
				prazosin	none	1	i.p.	0	30 min	none	FS	n.s.
				prazosin	none	2	i.p.	0	30 min	none	FS	n.s.
				prazosin	none	4	i.p.	0	30 min	none	FS	n.s.
				prazosin	none	0.25	i.p.	0	30 min	none	TS	n.s.
				prazosin	none	0.5	i.p.	0	30 min	none	TS	n.s.
				prazosin	none	1	i.p.	0	30 min	none	TS	n.s.
				prazosin	none	2	i.p.	0	30 min	none	TS	inc mob
				prazosin	none	4	i.p.	0	30 min	none	TS	inc mob
Stone and Quartermain (1999)	M	S-W	M	prazosin	none	0.5	i.p.	0	20 min	none	TS	inc imm
				prazosin	none	2	i.p.	0	20 min	none	TS	inc imm
Evangelista et al. (1987)	R	CD-COBS	M	prazosin	none	3	s.c.	0	90 min	none	FS	n.s.
Poncelet et al. (1987)	R	S-D	M	prazosin	desipramine	2, 32	i.p.	0	30 min	none	FS	inc imm
Cervo and Samanin (1991)	R	S-D	M	prazosin	none	3	s.c.	0	60 min	none	FS	n.s.
Detke et al. (1995)	R	S-D	M	prazosin	none	1	s.c.	2	60 min	none	FS	n.s.
				prazosin	8-OH-DPAT	1, 0.5	s.c.	2	60 min	none	FS	n.s.
Schreiber and De Vry (1993)	R	Wist	M	prazosin	none	0.1	i.p.	2	60 min	none	FS	n.s.
				prazosin	none	0.3	i.p.	2	60 min	none	FS	dec imm
				prazosin	8-OH-DPAT	0.1, 3	i.p.	2	60 min	none	FS	n.s.
				prazosin	8-OH-DPAT	0.3, 3	i.p.	2	60 min	none	FS	n.s.
Stone et al. (2011)	M	S-W	M	terazosin	none	1 nmol	i.c.v.	0	5 min	none	TS	inc imm
Wu et al. (2017)	R	S-D	M	benoxathian	none	5 μg	prelimbic infusion	0	10 min	none	FS	inc imm
				benoxathian	none	5 μg	prelimbic infusion	0	10 min	none	SP	dec sucr pref

*This table comprises mouse and rat studies from our literature search that used these drugs in the forced swim (FS), tail suspension (TS), or sucrose preference (SP) tests. The “Repeats” column indicates how many times a drug treatment was repeated in that group of animals, where zero repeats indicate a single administration of that drug or drug pair. The “Time Delay” column represents the amount of time between the last (or only) administration of the drug or pair of drugs and when the behavioral test was carried out. The “Stress” column indicates whether an acute or chronic stressor was administered prior to testing. The “Effect” column describes the type of statistically significant outcome in the behavioral test or otherwise shows that the result was not statistically significant (n.s.; *p* > 0.05). For single drug administration, the Effect column describes the effect relative to vehicle administration. For pairs of drugs, the Effect column compares the behavioral effect of the pair with when one drug alone was given in that experiment. All experiments that used a prior stressor are marked in red. Experiments that showed a statistically significant antidepressant-like effect are marked in green, whereas those with a depression-like effect are marked in blue. Other abbreviations: [Species: mouse (M), rat (R)], C57BL/6J (C57), Sprague-Dawley (S-D), Swiss Webster (S-W), Wistar (Wist), [Sex: male (M), female (F)], lipopolysaccharide (LPS), intraperitoneal (i.p.), subcutaneous (s.c.), intracerebroventricular (i.c.v.), once a day (q.d.), twice a day (b.i.d.), social defeat (soc defeat), maternal separation (mat sep), social isolation (soc isol), single prolonged stress (SPS), decreased immobility (dec imm), increased immobility (inc imm), increased mobility (inc mob), increased swimming (inc swim), increased climbing (inc clim), decreased sucrose preference (dec sucr pref)*.

## Alpha2 Agonists

Dating back several decades, there is a body of evidence suggesting that alpha2 adrenergic agonists such as clonidine and guanfacine, which inhibit the presynaptic release of NE and activate alpha2 receptors that are also located postsynaptically, have antidepressant-like properties in rodent models. While there are some opposing data suggesting that alpha2 *antagonists* can have antidepressant-like effects (Muguruza et al., 2013; Uys et al., 2017), a number of studies report that alpha2 agonists such as clonidine are therapeutic when administered acutely. A number of these studies indeed suggest that clonidine, by itself, can produce antidepressant-like effects in tests such as the FST (Malinge et al., 1988, 1989; Cervo and Samanin, 1991; Cervo et al., 1992; Asakura et al., 1993, 1994; Skrebuhhova et al., 1999; Masuda et al., 2001; O’Neill et al., 2001; Malikowska et al., 2017).

Clonidine, in many cases, when given at sub-effective doses, can also potentiate the antidepressant-like effects of a wide range of other drugs that have antidepressant properties such as SSRIs, NDRIs, TCAs, MAOIs, 5HT1A agonists, lithium, lamotrigine, and others (Malinge et al., 1988, 1989; Bourin et al., 1991, 1996; Bourin et al., 2002; Hascoät et al., 1991; Hascoet et al., 1994; Redrobe and Bourin, 1997, 1998; Skrebuhhova et al., 1999; Kaster et al., 2007; Zeidan et al., 2007; Taksande et al., 2009; Kotagale et al., 2013). In some cases these effects were shown to be counteracted by alpha2 antagonists such as idazoxan or yohimbine, suggesting clonidine achieves its antidepressant-like properties through activation of the alpha2 receptor (Malinge et al., 1988, 1989; Cervo and Samanin, 1991; Masuda et al., 2001; O’Neill et al., 2001; Zeidan et al., 2007).

In contrast to these potentially therapeutic properties of clonidine, it has also been suggested that this drug can promote depression-like behavior in rodents (Kitada et al., 1983; Parale and Kulkarni, 1986; Ferrari et al., 1991; Rénéric et al., 2002), or under some circumstances has no substantial effect either alone or when co-administered with other putative antidepressants (Kitada et al., 1983; Evangelista et al., 1987; Antkiewicz-Michaluk et al., 2017).

It has also been shown that molecular overexpression of alpha2C adrenoceptors can decrease immobility in the mouse FST (Sallinen et al., 1999), perhaps mimicking the antidepressant-like effects of alpha2 agonists such as clonidine. Antidepressant-like effects of two other alpha2 agonists, guanfacine and dexmedetomidine, have also been reported in rodent models (Stone et al., 2011; Mineur et al., 2015, 2018).

## Beta Blockers

Beta blockers such as propranolol and nadolol (non-selective beta1/2 antagonists), metoprolol and atenolol (beta1), and butoxamine (beta2) can exhibit antidepressant-like activity in the FST (Chopra et al., 1988; Beĭer, 1994; Aisa et al., 2008; Park et al., 2012; Zaidi et al., 2020), including potentiation of sub-effective doses of other putative antidepressants such as baclofen (Aley and Kulkarni, 1990), or antagonizing depression-like effects of other agents (Parale et al., 1987). A mouse study of propranolol and nadolol found that whereas these two drugs did not exhibit therapeutic effects in the TST, propranolol did show an antidepressant-like decrease in TST-induced hyperthermia (Liu et al., 2003). The non-selective beta blocker nebivolol has been shown to counteract the depression-like behavioral and pathophysiological effects of the chemotherapeutic agent cisplatin (Abdelkader et al., 2017). An immunocytochemical study of propranolol showed that it could reduce the number of cells that stained for Fos-like immunoreactivity in various subcortical and cortical regions, resembling standard antidepressants such as imipramine and desipramine (Duncan et al., 1996).

In contrast to these potentially therapeutic properties of beta blockers, it has also been suggested that these drugs can promote depression-like behavior in rodents (Abel and Hannigan, 1994; Stone and Quartermain, 1999; Al-Tubuly et al., 2008) including in the presence of other putative antidepressants (Zhang et al., 2009; Gu et al., 2012), or under some circumstances, they have no substantial effect either alone or when co-administered with other putative antidepressants (Danysz et al., 1986; Evangelista et al., 1987; Finnegan et al., 1987; Teste et al., 1990; Beĭer, 1994; Detke et al., 1995; Pesarico et al., 2014; Sekio and Seki, 2015; Zaidi et al., 2020). A number of studies also suggest that the beta3 *agonist* amibegron (also called SR58611A) has antidepressant-like properties in rodents (Consoli et al., 2007; Overstreet et al., 2008; Stemmelin et al., 2008, 2010; Tamburella et al., 2010), and it may achieve these effects by modulating serotonergic and noradrenergic signaling that is triggered by activation of beta3 receptors (Claustre et al., 2008).

## Alpha1 Antagonists

Alpha1 antagonists such as prazosin and benoxathian can also exhibit antidepressant-like activity in the FST or TST (Sekio and Seki, 2015; Kurosawa et al., 2016; Wu et al., 2017), including potentiation of other putative antidepressants such as imipramine (Al-Tubuly et al., 2008). In contrast, it has also been suggested that alpha1 antagonists can promote depression-like behavior (Stone and Quartermain, 1999; Al-Tubuly et al., 2008), including in the presence of other putative antidepressants or electroconvulsive therapy (ECT; Danysz et al., 1986; Poncelet et al., 1987; Teste et al., 1990; Kaster et al., 2007; Sugimoto et al., 2011; Gu et al., 2012; Ribeiro and Pupo, 2015). Under some circumstances they have no substantial depression-related behavioral effect either alone or when co-administered with other putative antidepressants (Evangelista et al., 1987; Malinge et al., 1988, 1989; Cervo and Samanin, 1991; Schreiber and De Vry, 1993; Detke et al., 1995; Sugimoto et al., 2011; Pesarico et al., 2014). In addition, mice expressing constitutively active mutant alpha1A (but not alpha1B) adrenoceptors exhibit antidepressant-like activity in the FST and TST, that is counteracted by prazosin (Doze et al., 2009).

Table 1 summarizes the results from, and experimental parameters used in the above rodent studies on noradrenergic transmission reducing drugs in the FST, TST, and sucrose preference test. A brief analysis of the table suggests a few prominent themes or findings: (1) clonidine is the drug with the most experimental evidence supporting an antidepressant-like role. Those data support its therapeutic-like role across a variety of both mouse and rat strains, in both the FST and TST, and an amplifying beneficial role when paired with a wide range of established antidepressants; (2) there is less support at this time of an antidepressant-like role for various beta blockers and the alpha1 antagonist prazosin, where a number of studies show depression-like effects for these drugs (although other data are supportive). These drugs appear to not have been studied as extensively in these tests as clonidine; (3) very few of the studies used female mice, which should be a priority in future studies, especially considering that the rate of MDD in women is approximately twice that in men (Baxter et al., 2014; Albert, 2015); (4) only a few of the studies used C57BL/6J mice, which are widely used in behavioral neuroscience, and could be combined with studies of additional strains of mice in further investigations; and (5) prior exposure to chronic stress, which can induce MDD in susceptible human subjects (Hosang et al., 2014; Bonde et al., 2016), was rarely used in these studies and should be further addressed with additional experiments.

## Discussion

The preclinical data reviewed above address the issue of whether noradrenergic transmission reducing pharmacological agents have antidepressant-like behavioral properties in rodents. While many of these studies, perhaps most numerously and convincingly for the alpha2 agonist clonidine, suggest that these drugs have therapeutic effects, a number of the publications found no effect or depression-like effects, including for the beta blocker propranolol and the alpha1 antagonist prazosin. How do we reconcile such opposing effects across studies for these drugs? Some possibilities are that they may be due to genetic differences across strains or species of animals, varying responses to acute or chronic stress, or in some cases different behavioral tests that were used. Another explanation is that since there may be a u-shaped or Janus-faced dose-response relationship for noradrenergic signaling (Arnsten, 2007; Giustino et al., 2016; Giustino and Maren, 2018), the different drug doses used in the above studies could have opposing behavioral effects, including through interaction with divergent cortical and subcortical circuits, which may vary across species and strain. If alpha2 agonists such as clonidine and guanfacine really do have more robust antidepressant-like properties than beta blockers and alpha1 antagonists, this may relate to the more general effect of alpha2 agonists decreasing the presynaptic release of NE (Gresch et al., 1995; Van Gaalen et al., 1997), which would in principle affect signaling at all subtypes of adrenoceptors simultaneously.

**Figure 1:**
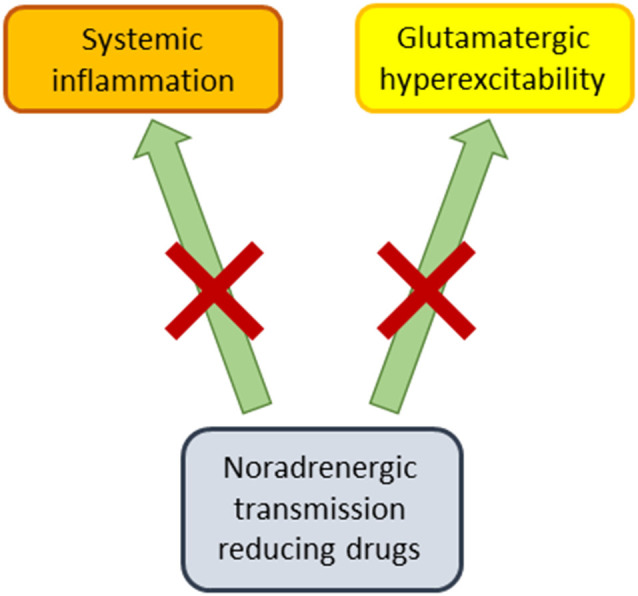
Proposed therapeutic mechanisms of noradrenergic transmission reducing drugs. These pharmacological agents (alpha2 agonists, beta blockers, alpha1 antagonists) may produce antidepressant-like effects by dampening systemic inflammation, while also counteracting glutamatergically-mediated neural hyperexcitability.

A number of the studies reviewed above investigated the interaction of noradrenergic transmission reducing agents with other types of drugs. Several of these studies suggest that these noradrenergic drugs can potentiate the antidepressant-like effects of SSRIs or 5HT1A agonists (Malinge et al., 1988, 1989; Bourin et al., 1991, 1996; Hascoet et al., 1994; Redrobe and Bourin, 1997, 1998; Taksande et al., 2009), although not all studies or data were supportive (Redrobe and Bourin, 1998; Rénéric et al., 2002). Despite these discrepancies, this may be a treatment strategy that has clinical ramifications for the pharmacotherapy of MDD in human subjects. It has been previously suggested (Dremencov et al., 2007a,b; Guiard et al., 2008; Fitzgerald and Watson, 2019) that serotonin and NE may have functionally opposed properties, which is consistent with the hypothesis that noradrenergic transmission reducing drugs can amplify the effects of SSRIs under some conditions. We also suggest here, consistent with a statement in our prior publication (Polis et al., 2019), that noradrenergic transmission reducing drugs may be antidepressants in a subset of humans suffering from MDD, who would also be responsive to the rapidly acting antidepressant ketamine, and to ECT. In this scenario, noradrenergic transmission reducing agents may interact with glutamatergic signaling to chronically suppress neural hyperexcitability associated with some cases of MDD (Figure 1), and possibly have rapid therapeutic onset like ketamine (Polis et al., 2019). While the molecular mechanisms through which noradrenergic transmission reducing drugs may achieve antidepressant-like effects are not well understood at this time, one possibility is that they selectively dampen certain intracellular signaling pathways after acting upon alpha and beta-adrenergic G protein-coupled receptors. There is already evidence, for example, that NE modulates the Ras/MAPK, PI3K/Akt, JAK/STAT pathways (Muthalif et al., 1998; Yanagawa et al., 2010; Guo et al., 2013; Maity et al., 2020).

One might argue that noradrenergic transmission reducing drugs are, based on monoaminergic theories of mood disorders, more likely to have mood-stabilizing than antidepressant properties. After all, beta blockers such as propranolol have historically been more associated with induction of MDD or depressive-like symptomatology (Koella, 1985; Rosen and Kostis, 1985) (but also see: Kim et al., 2019; Kessing et al., 2020), or with attenuation of hypomania or mania (Emrich et al., 1979; Nemeth and Mckenzie Chustz, 2020), where the latter property has also been attributed to clonidine (Hardy et al., 1986; Nemeth and Mckenzie Chustz, 2020). One possibility is that if these drugs really are antidepressants under some conditions, they achieve these effects in individuals who exhibit neural “decoupling” of NE with dopamine in mood-related circuits. In such an individual, elevated noradrenergic signaling may result in MDD rather than dopamine-facilitated hypomania or mania (Diehl and Gershon, 1992). Since MDD is also associated with systemic inflammation (Miller et al., 2009), noradrenergic transmission reducing agents may also produce antidepressant effects by counteracting neuroinflammation (Chen et al., 2015; Ding et al., 2019; Apple et al., 2020; Figure 1).

In conclusion, while there are conflicting data in rodents as to whether noradrenergic transmission reducing drugs have antidepressant-like properties, a number of studies reviewed above support this hypothesis, at least under some experimental conditions. At present, it is not clear whether neural noradrenergic transmission is elevated or suppressed in MDD (Waldmeier, 1981), where perhaps each state exists in different individuals. For these reasons, additional preclinical, mechanistic studies are needed, including those that induce depression-like behavior in animal models through the use of chronic mild stress. Based on the foundation of preclinical studies reviewed briefly here, further investigation of noradrenergic transmission reducing drugs in human mood disorders also appears warranted.

## Author Contributions

The author alone conceived of, researched, wrote, and edited this publication.

## Conflict of Interest

The author declares that the research was conducted in the absence of any commercial or financial relationships that could be construed as a potential conflict of interest.

## Publisher’s Note

All claims expressed in this article are solely those of the authors and do not necessarily represent those of their affiliated organizations, or those of the publisher, the editors and the reviewers. Any product that may be evaluated in this article, or claim that may be made by its manufacturer, is not guaranteed or endorsed by the publisher.
